# 

**Published:** 1879-04

**Authors:** 


### CONTENTS.

ORIGINAL.

ART. I.—Progress and Prospects of the Buffalo Medical College. A speech at the banquet of the Alumni Association in response to the toast of Alma Mata. By Prof. Jas. White, M. D., ........ 321

ART. II.—Warm and Hot Water as a Surgical Dressing. Resume of Cases. By Prof. Frank H. Hamilton, M. D., ........ 328

ART. III.—Early Indications of Insanity. By Judson B. Andrews, M. D., Utica, N. Y., .. 340

EDITORIAL.

Surgeon General Woodworth, ........ 353
Eye and Ear Infirmary. or Oculist and Aurist, . 353
Extract Malt and its Combinations, ........ 354
Maltine, ........ 354
Medicinal Wines, ........ 354
Buffalo Telephonic Exchange, ........ 354
Activity in Book-making, ........ 354
Religion and Chloroform, ........ 354

BOOKS REVIEWED:

Monograph on Diphtheria, ........ 355
Antagonism of Therapeutics, ........ 355
Localization in Diseases of the Brain, ..... 356
Index to Original Communications in Medical Journals of the United States and Canada, ........ 356
On Rest and Pain, ........ 357
Medical Chemistry, ........ 357
A guide to the Practical Examination of the Urine, ........ 357
An Atlas of Human Anatomy, ........ 358
Practical Surgery, including Ligations and Amputations, ........ 358
Transactions of the Medical Society, of the State of New York, ........ 358
Transactions of the Ohio Medical Society, . 358
Books Received, ........ 358
Pamphlets Received, ........ 359

THE BUFFALO

